# Hepatic steatosis is associated with dysregulated cholesterol metabolism and altered protein acetylation dynamics in chickens

**DOI:** 10.1186/s40104-023-00910-8

**Published:** 2023-08-12

**Authors:** Xiaoli Guo, Qianqian Zhou, Jiaming Jin, Fangren Lan, Chaoliang Wen, Junying Li, Ning Yang, Congjiao Sun

**Affiliations:** National Engineering Laboratory for Animal Breeding and Key Laboratory of Animal Genetics, Breeding and Reproduction, Ministry of Agriculture and Rural Affairs, China Agricultural University, Beijing, 100193 China

**Keywords:** Acetylation, Cholesterol metabolism, Hepatic steatosis, Laying hens, Multiomics

## Abstract

**Background:**

Hepatic steatosis is a prevalent manifestation of fatty liver, that has detrimental effect on the health and productivity of laying hens, resulting in economic losses to the poultry industry. Here, we aimed to systematically investigate the genetic regulatory mechanisms of hepatic steatosis in laying hens.

**Methods:**

Ninety individuals with the most prominent characteristics were selected from 686 laying hens according to the accumulation of lipid droplets in the liver, and were graded into three groups, including the control, mild hepatic steatosis and severe hepatic steatosis groups. A combination of transcriptome, proteome, acetylome and lipidome analyses, along with bioinformatics analysis were used to screen the key biological processes, modifications and lipids associated with hepatic steatosis.

**Results:**

The rationality of the hepatic steatosis grouping was verified through liver biochemical assays and RNA-seq. Hepatic steatosis was characterized by increased lipid deposition and multiple metabolic abnormalities. Integration of proteome and acetylome revealed that differentially expressed proteins (DEPs) interacted with differentially acetylated proteins (DAPs) and were involved in maintaining the metabolic balance in the liver. Acetylation alterations mainly occurred in the progression from mild to severe hepatic steatosis, i.e., the enzymes in the fatty acid oxidation and bile acid synthesis pathways were significantly less acetylated in severe hepatic steatosis group than that in mild group (*P* < 0.05). Lipidomics detected a variety of sphingolipids (SPs) and glycerophospholipids (GPs) were negatively correlated with hepatic steatosis (*r* ≤ −0.5, *P* < 0.05). Furthermore, the severity of hepatic steatosis was associated with a decrease in cholesterol and bile acid synthesis and an increase in exogenous cholesterol transport.

**Conclusions:**

In addition to acquiring a global and thorough picture of hepatic steatosis in laying hens, we were able to reveal the role of acetylation in hepatic steatosis and depict the changes in hepatic cholesterol metabolism. The findings provides a wealth of information to facilitate a deeper understanding of the pathophysiology of fatty liver and contributes to the development of therapeutic strategies.

**Supplementary Information:**

The online version contains supplementary material available at 10.1186/s40104-023-00910-8.

## Background

Fatty liver syndrome (FLS) often occurs in caged laying hens, especially during the later stages of the laying cycle, and poses a threat to the well-being and productivity of laying hens, and the primary hazards are as follows: (1) it is the main cause of noninfectious death of laying hens [1, 2]; (2) it affects the development of follicles, leading to a decline in production performance, egg production rate and egg quality [3]; and (3) it influences the endogenous synthesis and catabolism of bile acids and other steroid hormones, resulting in a reduction in nutrient absorption [4, 5]. In humans, it is known as non-alcoholic fatty liver disease (NAFLD), but has undergone a name change in 2020 as metabolic dysfunction-associated fatty liver disease (MAFLD) [6]. Hepatic steatosis is the initial manifestation of both FLS and MAFLD. Given that the liver is the primary organ for lipogenesis in humans and birds [7], chicken hepatic steatosis is an excellent model for studying MAFLD in human. Thus, it is imperative to clarify the potential regulatory mechanism of hepatic steatosis from a systemic perspective.

Hepatic steatosis is frequently accompanied by aberrant lipid metabolism [8–10]. The maintenance of lipid homeostasis is intricately dependent on the hepatic metabolic pathway, and the disruptions of lipid metabolism in turn can lead to the onset of hepatic steatosis, such as the high levels of free fatty acids (FFA), triglycerides (TG), cholesterol and other metabolites [11]. Therefore, alterations of metabolic signatures in the liver can be regarded as markers of distinct subtypes or as diagnostic biomarkers for hepatic steatosis in general. Irregular lipid metabolism has been identified to be affected by numerous proteins, mainly through alterations in protein expression, posttranslational modifications and protein interactions [12–14]. Among them, lysine (K) acetylation, as an evolutionarily highly conserved posttranslational modification mechanism (PTM), plays a crucial role in the development and progression of the diseases related to metabolism [15, 16]. In particular, acetylation has been reported to play a significant role in numerous metabolic pathways, including the regulation of gluconeogenesis, tricarboxylic acid (TCA) cycle, and fatty acid oxidation in the liver [17–19]. Therefore, there may be an interaction among lipid metabolism, protein expression and acetylation, which are involved in the regulation of hepatic steatosis. Integrated analysis of changes in transcriptome, proteome, acetylome, and lipidome under the context of hepatic steatosis can be a powerful strategy to characterize the relationship between highly connected molecular regulation and lipid content.

To date, the regulatory mechanism on hepatic steatosis in laying hens remains unclear, with only limited findings having compared changes in expression at the mRNA, protein, lncRNA and methylation levels in chickens with fatty liver [20–23]. The present study aimed to systematically investigate the molecular mechanisms of chicken hepatic steatosis with multiomic approaches.

## Materials and methods

### Ethics statement

The experiments were approved by the Animal Welfare Committee of China Agricultural University (permit no. AW32303202-1-1) and performed in accordance with the protocol outlined in the “Guide for Care and Use of Laboratory Animals” (China Agricultural University, Beijing, China).

### Animals and sample collection

A chicken population consisting of 686 female birds derived from Rhode Island Red breed in Beijing Huadu Yukou Poultry Breeding Co., Ltd., (China) was used in the current study. All chickens were reared in same conditions from hatching, and each chicken housed in an individual cage. The chickens were fed the same basic diet and had free access to feed and water. The illumination schedule followed a photoperiod of 16 h of light and 8 h of darkness on a daily basis (16L:8D). Hens were euthanized by cervical dislocation at 90 weeks of age. Half of each liver tissue was frozen in liquid nitrogen and immediately stored at −80 °C for subsequent sequencing and biochemical assays. The remaining liver tissue were fixed in formalin for 48 h for histological analysis.

### Histological analysis and evaluation of hepatic steatosis

Paraformaldehyde-fixed, paraffin-embedded livers were sectioned and stained with hematoxylin and eosin (H&E) staining reagent. Images of each liver section were obtained using a Canon EOS 7D digital camera (Canon, Tokyo, Japan). A total of 686 liver-stained sections were blindly evaluated by an experienced pathologist, and 90 individuals with the most prominent characteristics were selected for subsequent research. These 90 individuals could be clearly divided into three groups, as modified from Kleiner et al. [24]: HS0, healthy liver without lipid accumulation(< 5% steatosis of hepatocytes); HS1, mild fat accumulation in the liver (5%–33% steatosis of hepatocytes); HS2, massive fat accumulation (> 33% steatosis of hepatocytes). No fibrosis or hemorrhage was observed in the livers of any laying hens in this study.

### RNA sequencing and data analysis

A total of 90 liver samples (30 samples per group) were used for RNA-seq. Total RNA was extracted using the Eastep® Super Total RNA Extraction Kit (cat no: LS1040, Promega, Shanghai, China) following the manufacturer’s instructions. Transcriptome sequencing libraries were constructed according to the standard Illumina RNA-seq protocol and sequenced on the Illumina Novaseq platform (150 bp paired-end reads, PE150). Reads containing adaptor contamination, low quality bases, and undetermined bases were removed using Fastp (v.0.20.1) [25]. The clean reads were aligned to the chicken reference genome (GRCg6a) using HISAT2 (v.2.0.5) [26] with the default parameters. SAM files were converted to the BAM format using Samtools (v.1.11) [27]. Then, the reads were counted for each gene using featureCounts (v.1.6.3) [28]. Differentially expressed genes (DEGs) were identified with DESeq2 (v.1.32.0) [29] according to thresholds of an adjusted *P* value < 0.05 and |fold change| > 2.

### Proteomic and acetylomic analyses

Four samples were randomly selected from each group for the proteomic and acetylomic analysis. Samples were removed from −80 °C, well ground to powder with liquid nitrogen, and then lysed by ultrasonication. After removal of cell debris, the supernatant was collected, and the protein concentration was determined using a BCA kit (cat no: 23225, Thermo Scientific, Waltham, MA, USA). Equal amounts of protein from each sample were taken for digestion, and the precipitates were washed with 20% trichloroacetic acid (TCA) and prechilled acetone. After drying the precipitate, triethylammonium bicarbonate (TEAB) at a final concentration of 200 mmol/L was added, the precipitate was broken up by sonication, and trypsin (1:50) was added and digested overnight. Dithiothreitol (DTT) was added to a final concentration of 5 mmol/L and reduced for 30 min at 56 °C. Iodoacetamide (IAA) was added to a final concentration of 11 mmol/L, after which the samples were incubated for 15 min at room temperature while protected from light.

For proteomic analyses, tryptic peptides were solubilized in 0.5 mol/L TEAB. The TMT labeling reagent (cat no: 90068, Thermo Scientific, Waltham, MA, USA) was dissolved in acetonitrile, mixed with peptides, and incubated at room temperature for 2 h. Five microliters of each labeled sample were pooled, then desalted with Strata X C18 SPE column (Phenomenex, Torrance, CA, USA) and dried by vacuum centrifugation. The samples were fractionated by high pH reverse-phase HPLC using Agilent 300 Extend C18 column (5 μm particles, 4.6 mm ID, 250 mm length). Briefly, peptides were separated into 80 fractions using a gradient of 2% to 60% acetonitrile in 10 mmol/L ammonium bicarbonate pH 10 for 80 min, and peptides were pooled into 9 fractions and dried by vacuum centrifugation. Then, peptides were dissolved and separated using the EASY-nLC 1200 ultra high performance liquid chromatography (UHPLC) system (Thermo Scientific, Waltham, MA, USA), then added into the nanospray ion (NSI) source for ionization and then analyzed by the Q Exactive™ HF-X mass spectrometer (Thermo Scientific, Waltham, MA, USA). The electrospray voltage applied was 2.0 kV. For the scan range of 350–1,600 *m/z*, the full MS scan resolution was set to 60,000. Up to the 20 most abundant precursors were then selected for further MS/MS analysis with 30 s dynamic exclusion. The higher energy collisional dissociation fragmentation was performed at a normalized collision energy (NCE) of 28%. These fragments were detected in Orbitrap at a resolution of 30,000. Fixed first mass set to 100 *m/z*. The automatic gain control (AGC) target was set to 100,000, the intensity threshold was 33,000, and the maximum injection time was 60 ms.

For acetylomic analyses, the pre-washed acetylated beads (cat no: PTM-104, Jingjie PTM BioLab, Hangzhou, China) were added and incubated at 4 °C for modification enrichment. After incubation, the peptide bound to the beads was eluted, and then the eluate was collected and vacuum dried. The peptides were desalted with C18 ZipTips (Merck Millipore, Darmstadt, Germany) and vacuum freeze dried for liquid chromatography‒mass spectrometry (LC‒MS) analysis. The peptides were dissolved and separated by the NanoElute UHPLC system (Bruker Daltonics, Bremen, Germany), then injected into the capillary ion source for ionization and then analyzed by timsTOF Pro mass spectrometer (Bruker Daltonics, Bremen, Germany). The electrospray voltage applied was 1.6 kV, and the peptide precursor ions and their secondary fragments were detected and analyzed using high-resolution TOF. The scanning range of MS/MS was set at 100–1,700. The data acquisition mode uses the Parallel Accumulation Serial Fragmentation (PASEF) mode. After a primary mass spectrometer was collected, 10 times of PASEF mode was used to collect the secondary spectrum of the precursor ion charge number in the range of 0–5. The dynamic exclusion time of the tandem mass spectrometry scan was set to 30 s to avoid repeated scanning of the precursor ion.

The resulting data were processed using the MaxQuant search engine (v.1.6.15.0) [30]. Tandem mass spectra were searched against the Gallus_gallus database (27,535 entries) concatenated with a reverse decoy database. Carbamidomethyl on Cys was specified as a fixed modification, and acetylation on the protein N-terminus and oxidation on Met were specified as variable modifications. The FDR was adjusted to < 1%.

### Enrichment analysis

The Metascape online tool (http://metascape.org) was used to annotate genes. Eggnog-mapper (v.2.0) [31] was used to annotate proteins. For each protein sequence, the result with the highest score in the BLAST alignment is selected for annotation. The gene ontology (GO) annotations were based on categories of biological process, cellular component, and molecular function. The Kyoto Encyclopedia of Genes and Genomes (KEGG) database was used to annotate pathways. *P* < 0.05 was set as the threshold for significant enrichment.

### Soft clustering of protein expression

The R package “Mfuzz” (v.2.52.0) [32] was used to cluster the proteins with the same expression patterns. Pathway enrichment analysis was performed for the proteins in each cluster separately. Cluster membership was visualized by a heatmap using the “heatmap.2” function of the R package “gplots” (v.3.1.1).

### Protein‒protein interaction (PPI) network

PPI analysis was performed using STRING (v.11.0) [33]. Functional protein association networks with confidence scores ≥ 0.7 (high confidence) were retained and visualized using the R package "networkD3" (v.0.4) [34].

### Lipid sample preparation and lipidomic assay

Six replicates were randomly selected from each group of RNA-seq analysis samples for the lipidomic analysis. Each group contained at least three samples that are the same as the proteomics and acetylomics analysis. Lipid contents were extracted from livers and detected by MetWare (http://www.metware.cn/). The sample was thawed on ice, approximately 20 mg homogenized with a steel ball in 1 mL of a mixture that consisting mainly of methanol, methyl tert-butyl ether (MTBE), and an internal standard mixture. After removing the steel ball, the mixture was whirled for 15 min. Then, 200 μL of water was added and the mixture was whirled for 1 min. Afterward, the mixture was centrifuged at 12,000 r/min and 4 °C for 10 min, and 300 μL of the supernatant was extracted and concentrated. The resulting powder was dissolved in 200 μL of reconstituted solution and stored at −80 °C, before being taken into the sample bottle for LC–MS/MS analysis. The sample extracts were analyzed using an ultra performance liquid chromatography (UPLC) (ExionLC™ AD, SCIEX, Framingham, MA, USA) and tandem mass spectrometry (MS/MS) (QTRAP® 6500+ , ABsciex, Los Angeles, CA, USA). In brief, a reversed phase Thermo Accucore™ C30 column (2.6 μm, 2.1 mm × 100 mm ID) was used at 45 °C. The gradient elution consisting of mobile phase A (10 mmol/L ammonium formate and 0.1% formic acid in 60% acetonitrile/water) and mobile phase B (10 mmol/L ammonium formate and 0.1% formic acid in 90% propan-2-ol/water) was applied. The column temperature was set to 55 °C, and the injection volume was 2 μL. The effluent was alternately connected to an ESI triple quadrupole linear ion trap (QTRAP)-MS, followed by LIT and triple quadrupole (QQQ) scans. The system was equipped with an ESI Turbo ion spray interface, operating in positive and negative ion modes. The ESI source operation parameters were as follows: ion source, turbo spray; source temperature 500 °C; ion spray voltage (IS) 5,500 V (Positive), −4,500 V(Neagtive); Ion source gas 1 (GS1), gas 2 (GS2), curtain gas (CUR) were set at 45, 55, and 35 psi, respectively. Instrument tuning and mass calibration were performed with 10 and 100 μmol/L polypropylene glycol solutions in QQQ and LIT modes, respectively. Based on the MWDB database (Metware database), the substances were quantified using the multiple reaction monitoring mode (MRM) of the triple quadrupole mass spectrometer.

The identified metabolites were annotated and mapped to the pathway database using the KEGG database (http://www.kegg.jp/kegg). Significantly regulated metabolites between groups were determined by variable importance of the projection (VIP ≥ 1) and |log_2_ fold change| ≥ 1. VIP values were generated from the OPLS-DA results using the R package MetaboAnalystR (v.3.2) [35]. The data were log-transformed (log_2_) and mean-centered before OPLS-DA. To avoid overfitting, a permutation test (200 permutations) was performed.

### Liver biochemical assays

Liver biochemical assays were performed according to the manufacturer’s instructions (Nanjing Jiancheng Bioengineering Institute, Nanjing, China). The concentrations of triglyceride (cat no: A110-2-1), free fatty acid (cat no: A042-2-1), total bile acid (TBA, cat no: E003-2-1), cholesterol (cat no: A111-1-1) and high-density lipoproteins (HDL, cat no: A112-1-1) were measured by colorimetric methods based on protein quantification (cat no: A045-2), while the concentration of very low-density lipoproteins (VLDL) were measured using an ELISA kit (cat no: H249). Thirty biological replicates (same as the RNA-seq samples) were performed for TG, FFA, TBA and cholesterol tests. Ten biological replicates (randomly selected from each hepatic steatosis group, and each group contained at least 4 samples that are the same as the lipidomic analysis) were performed for HDL and VLDL.

## Results

### Hepatic lipid accumulated with the severity of hepatic steatosis

**Fig. 1:**
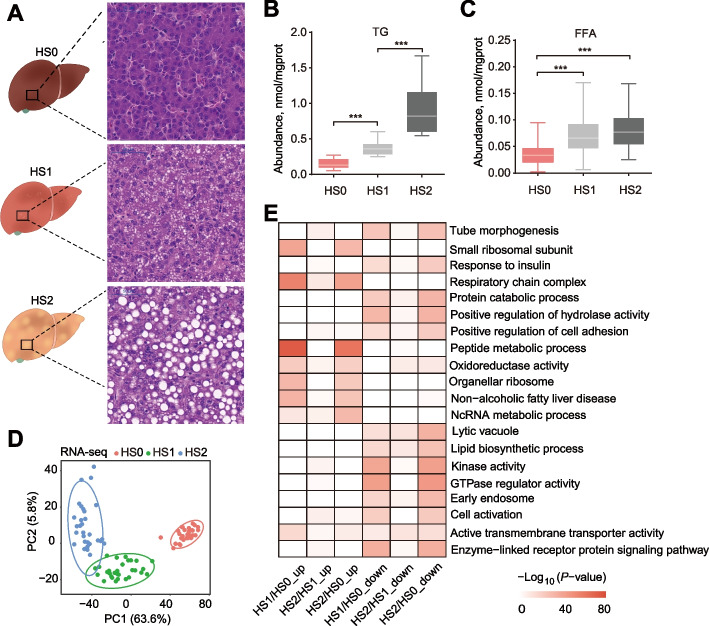
Hepatic lipid accumulation increased in laying hens with hepatic steatosis. **A** Histologic sections with H&E staining of livers from laying hens in different groups. **B** and **C** The concentrations of TG (**B**) and FFA (**C**) in different groups. The values are the mean ± SEM, *n* = 30 per group. * represents *P* < 0.05, ** represents *P* < 0.01 and ***represents *P* < 0.001. **D** PCA plot for RNA-seq. The points represent biological replicates. **E** Pathways of enrichment analysis with up- and down-regulated DEGs between different groups

We divided 686 individuals into three groups, about 217 individuals had healthy liver, 265 individuals had mild hepatic steatosis, and 204 individuals had severe hepatic steatosis. In each group, 30 individuals with the most prominent characteristics were selected for subsequent research, and each group had typical histological features, i.e., HS0 (*n* = 30) corresponded to the control group, with almost no lipid droplets in liver tissue. HS1 (*n* = 30) corresponded to the mild hepatic steatosis group, with small lipid droplets scattered in liver tissue and occasional large lipid droplets. HS2 (*n* = 30) corresponded to the severe hepatic steatosis group, enriched with large lipid droplets evenly dispersed in liver tissues (Fig. 1A). To verify the grouping accuracy, the TG and FFA in liver were quantified by biochemical assays. Consistent with the histology results, the TG content exhibited a significant increase with the severity of hepatic steatosis (*P* < 0.001, Fig. 1B). The hepatic concentration of FFA also showed an increasing pattern, with significantly higher abundance of FFA in hepatic steatosis groups (HS1 and HS2) than control group (*P* < 0.001, Fig. 1C).

Ninety liver tissues from one control and two hepatic steatosis groups (30 samples per group) were used for RNA-seq and transcriptomic analysis. Gene expression-based principal component analysis (PCA) revealed clear separation among groups, indicating high reproducibility of the transcriptomic profile of hepatic steatosis, and hepatic steatosis severity differentiated along the PC1 direction with 63.6% of explained variance (Fig. 1D). To identify DEGs, we performed pairwise comparative analysis among these three groups, and screened a total of 6,181 DEGs. Specifically, 3,407, 1,943 and 5,725 DEGs were identified between HS0 and HS1, between HS1 and HS2 and between HS0 and HS2, respectively (Additional file 1: Table S1, Additional file 2: Fig. S1A). The DEGs between HS0 and HS2 accounted for the majority of the entire DEG set (Additional file 2: Fig. S1B), suggesting dynamic transcriptional changes from control to the severe hepatic steatosis group. The unsupervised hierarchical clustering of all DEGs showed a distinct expression pattern among three groups (Additional file 2: Fig. S1C). Some DEGs were enriched in lipid metabolic terms, such as the non-alcoholic fatty liver disease and lipid biosynthetic process (Fig. 1E), which confirmed the rationality of hepatic steatosis grouping. We also identified that the expression of some genes in response to insulin pathway were changed significantly (Fig. 1E). Insulin plays a central role in regulation of lipid metabolism [36], suggesting the dysregulated insulin response in hepatic steatosis groups.

### Acetylation modification involved in maintaining the metabolic balance in the liver

**Fig. 2:**
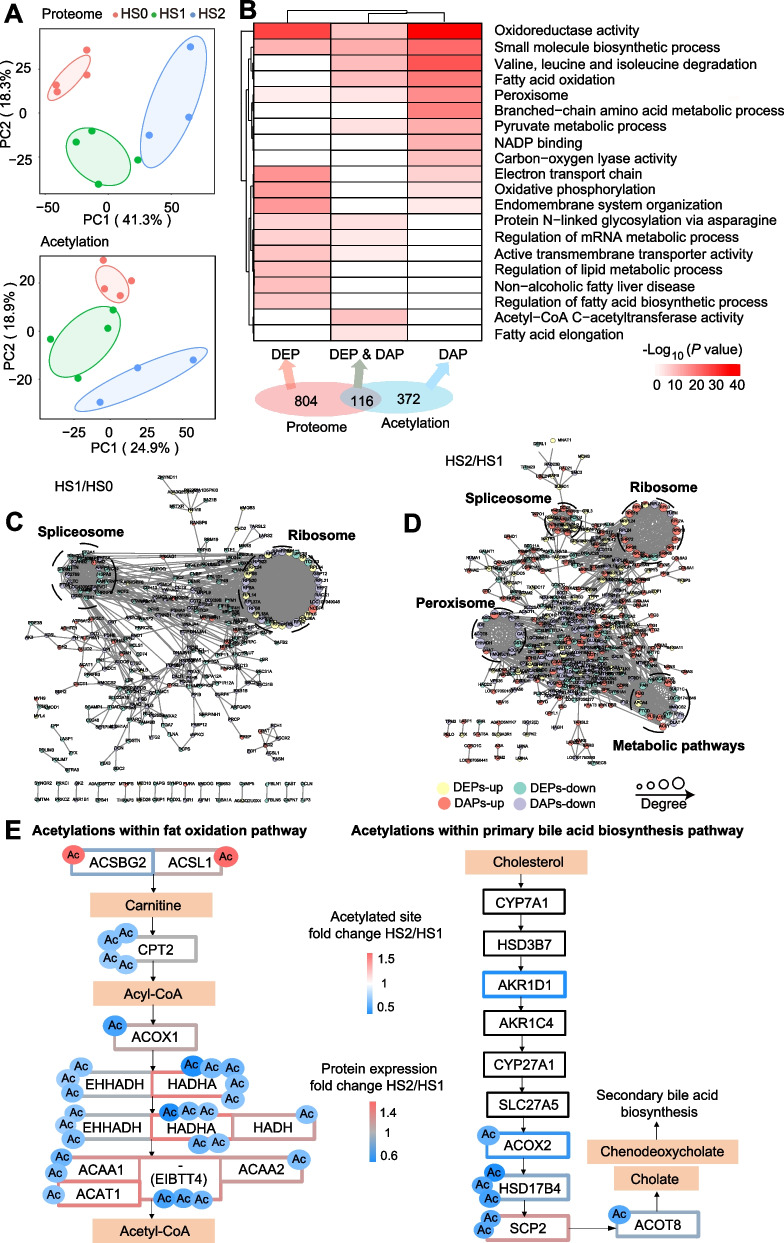
Proteome profiling analysis of different groups. **A** PCA plot of proteomics (upper) and acetylation (lower). The points represent biological replicates. **B** Enrichment analysis of DAPs and DEPs. **C** and **D** PPI network of DEPs and DAPs between HS0 and HS1 (**C**) and between HS1 and HS2 (**D**). The green circles represent the downregulated DEPs, the yellow circles represent the upregulated DEPs, the purple circles represent the hypoacetylated proteins, and the red circles represent the hyperacetylated proteins. **E** The fat oxidation and primary bile acid biosynthesis pathways were hypoacetylated in the HS2 group compared with the HS1 group. Proteins are labeled by gene symbols with identified acetylation sites. Boxes indicate protein expression (black indicates no significant change in protein expression), circles indicate acetylation sites, and the color scale designates fold-change (HS2/HS1 comparison for protein expression or acetylated site)

Since the number of DEGs was huge, the further investigation was performed from proteome and acetylome to identify candidate genes more accurately. In total, 5,967 proteins and 7,319 acetylated sites were quantified after filtration. The PCA plots showed clear separation between the control (HS0) and hepatic steatosis groups (HS1 and HS2) in both protein expression and acetylation (Fig. 2A). Further analysis revealed 920 DEPs and 707 differentially acetylated sites in 488 proteins (Additional file 1: Table S2 and S3, Additional file 2: Fig. S1D and E). The greatest number of DEPs occurred between HS0 and HS2, which is consistent with DEGs (Additional file 2: Fig. S1F). Given that protein acetylation is an important determinant of protein function and interaction, we compared the 488 DAPs between each of two groups. The number of acetylated sites and DAPs was greatest between HS1 and HS2 (Additional file 2: Fig. S1G), suggesting that protein acetylation may contribute to the development from mild to severe hepatic steatosis.

To categorize and characterize the proteins that underwent dynamic changes during the hepatic steatosis, we compared the functional enrichments of DEPs and DAPs (Fig. 2B). The terms, including regulation of fatty acid biosynthetic process, regulation of lipid metabolic process and NAFLD, were mainly enriched for DEPs without acetylation modification. The terms related to the DAPs without alteration in expression were mainly enriched in branched chain amino acid metabolic process and NADP binding. In contrast, significant terms, such as fatty acid elongation, fatty acid oxidation and oxidoreductase activity were enriched for DEPs and DAPs (Fig. 2B, Additional file 1: Table S4). Overall, both DEPs and DAPs were involved in the regulation of lipid metabolism. Subsequently, the PPI analysis was performed for DEPs and DAPs, and found extensive interactions between DEPs and DAPs. Except for spliceosomes and ribosomes, two highly connected clusters including metabolic pathways and peroxisome, were identified between HS1 and HS2 (Fig. 2C). In addition, oxidative phosphorylation and metabolic pathways were identified between HS0 and HS2 (Fig. 2D, Additional file 3: Fig. S2A). In general, peroxisome and oxidative phosphorylation are associated with metabolic pathways [37, 38], which means that DEPs interacted with DAPs to play a role in maintaining the metabolic balance in the liver.

### Acetylation promotes the progression from mild to severe hepatic steatosis

To better assess the regulatory role of acetylation in hepatic steatosis, a soft clustering analysis was performed on all quantified acetylated proteins. In total, eight clusters were identified based on distinct expression patterns among HS0, HS1, and HS2 groups (Additional file 3: Fig. S2B). Interestingly, the majority of acetylated proteins, whose expression were significantly altered between HS1 and HS2 (but may not have differed between HS0 and HS1), were involved in lipid metabolic pathways, including fatty acid degradation, arachidonic acid metabolism, fat digestion and absorption, peroxisome and peroxisome proliferator-activated receptor (PPAR) signaling (Additional file 3: Fig. S2B, except clusters 4 and 7). Therefore, we speculate that protein acetylation may contribute to the progression from mild to severe hepatic steatosis. Upon estimating the relative levels of acetylation of all peptides within lipid metabolism pathways between HS1 and HS2, we found that the fat oxidation pathway and the primary bile acid biosynthesis pathway had significantly lower acetylation in the HS2 group than in the HS1 group (Fig. 2E). Some important acetylated enzymes such as CPT2, EHHADH, HADHA, ACAA1, HSD17B4 and SCP2 have multiple significant modification sites. Given that lipid oxidation and primary bile acid synthesis are important biological processes of lipid metabolism in the liver, we preliminarily hypothesized that the dysfunction caused by acetylation modification of these proteins may have hampered metabolic processes. Additionally, these acetylated proteins are highly similar to the targets of mitochondrial lysine deacetylase sirtuin 3 (SIRT3) [39]. HADHA is a substrate widely regulated by SIRT3 [40], and its 5 acetylation sites were significantly differentially acetylated among three hepatic steatosis groups (*P* < 0.05, Fig. 2E). MDH2 is also reported to be a major SIRT3 target [41], and K306 in MDH2 was differentially acetylated between the HS1 and HS2 groups (*P* = 0.018, Additional file 3: Fig. S2C). In this context, we speculate that SIRT3 may play a role in mediating acetylation differences in this study. However, the expression of SIRT3 was not significantly different among the hepatic steatosis groups by proteomic analysis (Additional file 3: Fig. S2D).

### Dysregulation of cholesterol metabolism was associated with altered protein acetylation

**Fig. 3:**
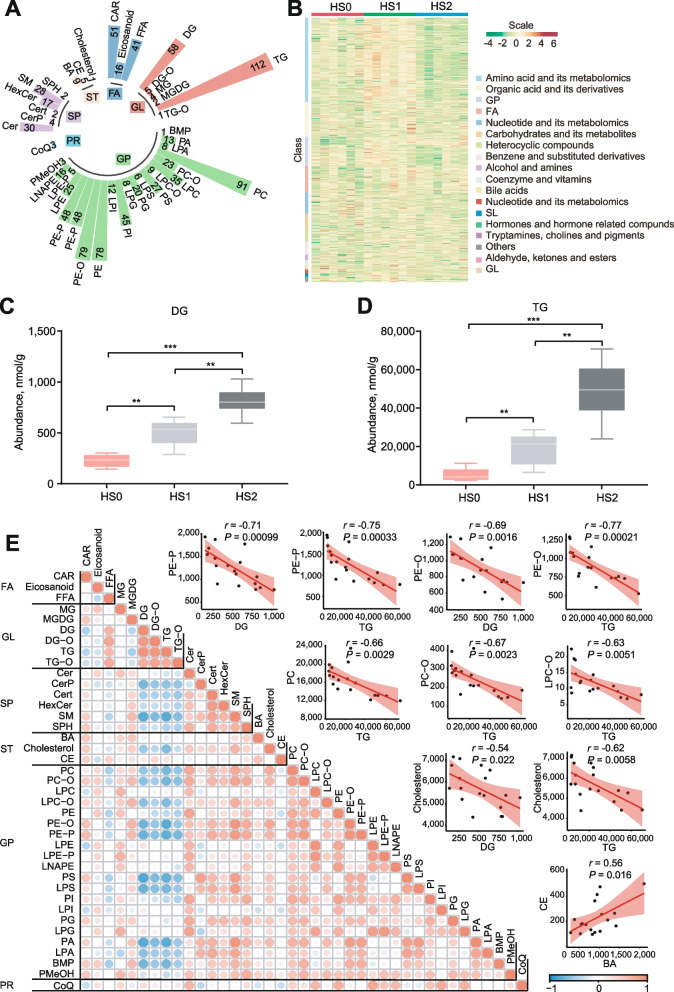
Identification of lipids in different groups. **A** Composition of lipid classes that were considered for subsequent analysis in all samples. **B** Heatmap of different lipids via hierarchical cluster analysis. Different rows correspond to different lipids, and red and green strips represent increased or decreased lipids, respectively. **C** Content of DG in different groups by lipidomic analysis. **D** Content of TG in different groups as determined by lipidomic analysis. **E** Heatmap and scatterplot of lipid classes (Pearson correlation). The values are the mean ± SEM. * represents *P* < 0.05, ** represents *P* < 0.01 and *** represents *P* < 0.001

Proteome and acetylome analysis proved that hepatic steatosis was accompanied by aberrant lipid metabolism. In order to obtain the specific lipids, we performed lipidomics to measure the lipid composition and quantity in different hepatic steatosis groups. A total of 943 lipids were identified, organized into 6 categories comprising 40 lipid classes [42, 43] (Fig. 3A, Additional file 1: Table S5). The abundance of 365 lipids changed significantly among hepatic steatosis groups (Fig. 3B). As expected, the contents of diglycerol (DG) and TG increased significantly with the severity of hepatic steatosis (Fig. 3C, D), which was consistent with the results of the biochemical assays (Fig. 1B). TG accumulation in the liver is a typical feature of hepatic steatosis, hence the correlation analysis was performed on all lipid classes to identify the lipids that significantly associated with the changes of DG and TG. The correlation analysis showed that the levels of DG and TG were negatively correlated with a variety of SPs and GPs (Fig. 3E), among which the decreased phosphatidylcholine (PC) and phosphatidylethanolamine (PE) have been proven to cause hepatic steatosis [44, 45].

**Fig. 4:**
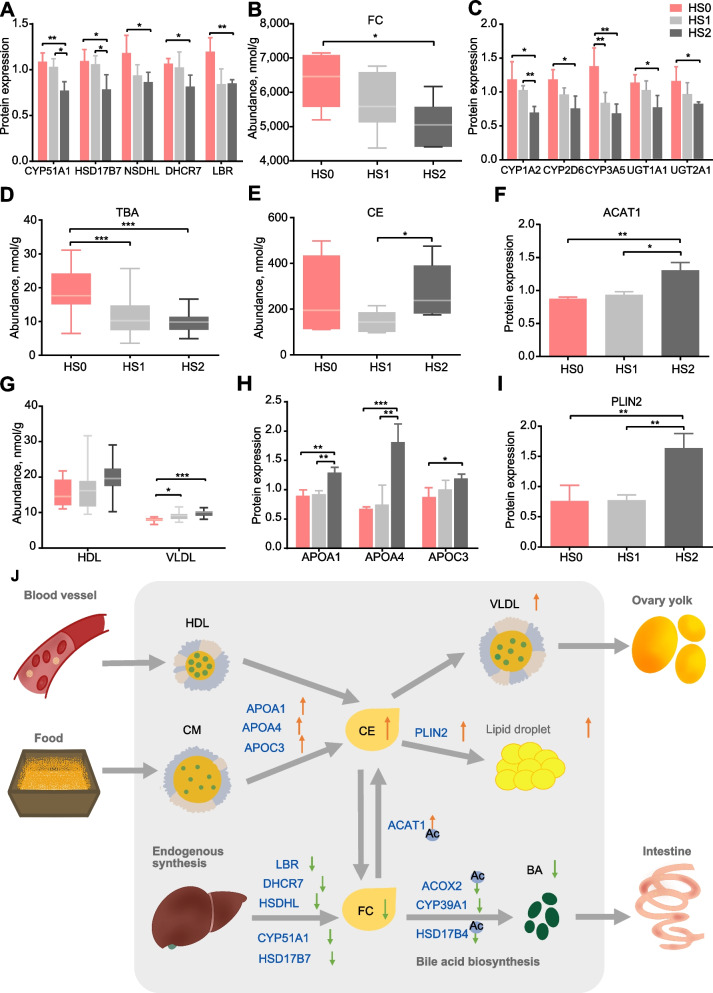
Lipid content and enzyme expression in the cholesterol metabolism pathway were altered in the hepatic steatosis group. **A** and **B** The expression of enzymes within the cholesterol synthesis pathway (**A**) and free cholesterol content (**B**) decreased with the severity of hepatic steatosis. **C** and **D** The expression of enzymes in the steroid hormone synthesis pathway (**C**) and total bile acid content (**D**) decreased with the severity of hepatic steatosis. **E** and **F** The content of CE (**E**) and the expression of ACAT1 (**F**) were increased in the HS1-HS2 stage. **G** and **H** The levels of cholesterol transport-related lipoproteins (**G**) and apolipoproteins (**H**) were increased with the severity of hepatic steatosis. **I** The expression of PLIN2 was increased with the severity of hepatic steatosis. The bar graphs indicate protein expression, and the box plots indicate metabolite content. The values are the mean ± SEM. * represents *P* < 0.05, ** represents *P* < 0.01 and *** represents *P* < 0.001. **J** Schematic of hepatic cholesterol metabolism in laying hens with severe hepatic steatosis. The shaded area indicates within the liver. Blue words indicate proteins, black words indicate lipids, and arrows indicate changes in protein expression or lipid content

Cholesterol was also negatively correlated with DG and TG (*r* ≤ −0.54, *P* < 0.05). Cholesterol is an important material for the synthesis of bile acids, and we demonstrate that the acetylation of important enzymes in the bile acid synthesis pathway was significantly reduced. And cholesterol esters (CE), the storage form of cholesterol, were positively correlated with bile acids (*r* = 0.56, *P* = 0.016; Fig. 3E). Subsequently, the combination analysis of proteomic and lipidomic were performed to systematically understand their direct relationship. It was observed that the expression of several enzymes in the cholesterol synthesis pathway gradually decreased with the severity of hepatic steatosis (Fig. 4A), and the free cholesterol (FC) quantified by colorimetric methods also gradually decreased (Fig. 4B). Cholesterol is an essential component for the synthesis of bile acid and other steroid hormones, and the expression of key enzymes for bile acid synthesis was decreased with the severity of hepatic steatosis (Fig. 2D), as did the expression of enzymes for steroid hormone synthesis (Fig. 4C). Among them, the altered expression of ACOX2, HSD17B4, SCP2 and ACOT8 were inextricably linked to acetylation modifications (Fig. 2D). In accordance with this, the content of total bile acid decreased significantly according to colorimetric methods (Fig. 4D). However, the content of CE increased significantly from HS1 to HS2 (Fig. 4E), as did the CE/FC ratio (Additional file 3: Fig. S2E). This may be due to a significant increase in acetyl-CoA acetyltransferase 1 (ACAT1) expression from HS1 to HS2, which is a membrane-bound protein that utilizes long-chain fatty acyl-CoA and cholesterol as substrates to form cholesteryl esters [46] (Fig. 4F). Excess cholesterol from the blood transported into the liver in the form of HDLs and exogenous cholesterol in the form of chylomicrons (CMs), while the cholesterol in the liver is transported out mainly through VLDLs [47]. By ELISA analysis, the lipoprotein contents of VLDLs were higher in the hepatic steatosis groups, but HDLs did not change significantly (Fig. 4G). Lipoproteins are composed of lipids, cholesterol and apolipoproteins, and the expression of apolipoproteins A1, A4 and C3 (APOA1, APOA4 and APOC3) increased significantly in the hepatic steatosis groups (Fig. 4H), suggesting an increased level of cholesterol transport. We also found that the expression of the well-known lipid droplet protein perilipin 2 (PLIN2) was significantly increased (Fig. 4I), which could promote deposition of the excess cholesterol in the lipid droplets. Based on these results, we propose that cholesterol synthesis, transport and secretion were aberrant in the liver of laying hens with severe hepatic steatosis (Fig. 4J).

## Discussion

Fatty liver is a complex trait caused by multiple factors, such as genetic, insulin resistance, hormone, nutritional, gut microbiota and epigenetic factors. All these factors contribute to hepatic steatosis in an intricate and interrelated manner [11, 48, 49]. Therefore, a systematic strategy is needed to resolve the mechanisms of hepatic steatosis. Here, we used a multiomics approach to analyze the changes in gene expression, proteins expression, acetylation modification and lipid metabolism at different stages of hepatic steatosis. The present work provides unique insights into the regulatory mechanisms underlying hepatic steatosis in laying hens, and prove beneficial for investigations pertaining to human MAFLD.

Hepatic steatosis develops when the formation of FAs (exogenous uptake and endogenous synthesis) in the liver is greater than the release of FAs (FA oxidation and VLDLs output); dysregulation of metabolism in any of these processes may cause the development of hepatic steatosis [8, 48]. Through transcriptomics, we identified numerous genes that were significantly differentially expressed between different hepatic steatosis groups. Most of these genes were involved in processes related to lipid synthesis, lipid transport and lipid degradation, demonstrating that hepatic steatosis development is accompanied by multifaceted disorders of lipid metabolism. This was confirmed by the results of proteome and acetylation analyses.

DAPs were specifically enriched in multiple metabolic processes. In fact, almost every enzyme in the TCA cycle, glucose metabolism and lipid metabolism process is acetylated in human liver tissue, and enzymes involved in intermediate metabolism are preferentially acetylated [50]. In recent years, accumulated research has shown that protein acetylation is involved in the regulation of the pathogenesis of fatty liver [18, 51]*.* Moreover, mitochondria are crucial for cellular energy metabolism processes, and acetylation modifications located in mitochondria play a crucial role in disorders of energy metabolism [52]. Mitochondrial acetylation is mainly controlled by the enzymatic activity of the NAD^+^-dependent deacetylase SIRT3, which has been shown to regulate metabolic pathways including fatty acid oxidation, ketogenesis, amino acid catabolism, and TCA cycles [39, 53–56]. Although the acetylation status of many substrates of SIRT3 was significantly altered in the hepatic steatosis group in this study, the expression of SIRT3 did not explain these changes. Rather, the dynamic deacetylation may have been due to metabolic disturbances, increases in mitochondrial NAD^+^ and activation of SIRT3 [57, 58]. We also found that some proteins contained multiple acetylation sites, such as HADHA and EHHADH. Both of them catalyze two steps of fatty acid oxidation and have been reported to be significant regulatory factors through acetylation in the development of fatty liver disease in dairy cows [18]. Interestingly, acetylation occurs mainly in the mild hepatic steatosis group, when there is already a certain degree of fatty acid accumulation in the liver. Consistent with this, the addition of fatty acids could increase the acetylation of EHHADH and change its activity [50]. Overall, we speculated that the elevated fatty acid concentrations in the mild hepatic steatosis group changed the acetylation statuses of metabolic enzymes in several important pathways, leading to the changes in the expression or activity of these enzymes and enforcing the condition.

In chickens, changes in food composition, such as high-fat, or high-energy, low-protein diets can induce fatty liver [59, 60]. A more recent study showed that low-choline diet can be utilized to rapidly generate a fatty liver model [61]. The liver is the primary organ responsible for choline metabolism, where it is found primarily as PC [62], produced by conversion of PE. We found that the levels of PC and PE were significantly negatively correlated with TG. It has been reported that the deficiency of hepatic PC reduces VLDL secretion, resulting in blocked TG transport [45, 63]. Thus, metabolic disorder of PC or PE in the liver can increase TG deposition and induce hepatic steatosis. In addition, PLIN2, a well-known lipid droplet protein, seems to correlate with hepatic lipid accumulation [64]. *Plin2*-deficient mice have been reported to have reduced TG content and to be protected against fatty liver development [65, 66]. The expression of PLIN2 was significantly increased in the severe hepatic steatosis group, which means that PLIN2 can mediate the increase in TG content to cause the occurrence of hepatic steatosis in laying hens. PLIN2 can also negatively regulate the secretion of VLDLs [67]. Like lipids, cholesterol is also an important component of VLDLs. In this context, although excess TG and cholesterol in the liver can combine with apolipoprotein to form VLDLs, the secretion of VLDLs is blocked, resulting in the accumulation of hepatic VLDLs, lipids and cholesterol.

Cholesterol homeostasis is an important factor for liver health, and elevated liver cholesterol can induce hepatic steatosis [61, 68]. Cholesterol is one of the basic components of cell membranes and is an essential precursor for the synthesis of bile acids and steroid hormones [69]. In this study, hepatic cholesterol metabolism was altered in laying hens with hepatic steatosis. Specifically, the synthesis of cholesterol and bile acid decreased significantly as the degree of hepatic steatosis increased. Bile acids have a positive effect on lipid metabolism, emulsifying fats, promoting the hydrolysis activity of lipase and lipoprotein esterase on fat, and transporting fats in the intestine to promote fat absorption [4]. Reduced bile acid secretion is an etiology of fatty liver [70–72]. Therefore, the decrease in endogenous cholesterol and bile acid synthesis may affect the digestion and absorption of lipids, promoting the development of hepatic steatosis. Another important aspect of maintaining cholesterol dynamic balance is the transport of intracellular cholesterol. Excess cholesterol from the blood and endogenous cholesterol from food bind to lipoproteins and are transported into the liver as HDLs and CMs [47, 68]. The levels of apolipoproteins (APOA and APOC) involved in these processes were significantly elevated in the hepatic steatosis group in this study, indicating an increase in exogenous cholesterol transport. Endogenous and exogenous cholesterol enter the bile acid synthesis pathway or are esterified to form neutral CE [73]. CE can be stored in liver lipid droplets or assembled with phospholipids and apolipoproteins to form VLDLs, which is subsequently secreted into the blood and eventually transported to the yolk for deposition [69, 74]. ACAT1 is the key enzyme catalyzing the synthesis of CE at the last step. More importantly, it is at the crossroads of glycolysis, fatty acid degradation, tryptophan metabolism, BCAA degradation, and the TCA cycle [75]. We found that ACAT1 expression was significantly increased in the severe hepatic steatosis group, possibly due to its reduced acetylation modification. This is supported by a previous report demonstrating that acetylation of several lysine sites in ACAT1 decreases ACAT1 activity [76].

## Conclusions

The development of hepatic steatosis is accompanied by multifaceted disorders of lipid metabolism, where elevated fatty acid concentrations can alter the acetylation statuses of enzymes in metabolic pathway and promote hepatic steatosis. Furthermore, the severity of hepatic steatosis was associated with a decrease in cholesterol and bile acid synthesis and an increase in exogenous cholesterol transport. The blockade of VLDLs secretion caused the accumulation of hepatic lipids and cholesterol, which promoted the development of hepatic steatosis. The framework of the multiomics approach offer a distinct new perspective for elucidating the pathogenesis and mechanism of hepatic steatosis in laying hens.

### Supplementary Information

**Additional file 1:** **Table S1.** Differentially expressed genes identified by RNA-seq. **Table S2.** Differentially expressed proteins identified by the proteome. **Table S3.** Differentially acetylated sites identified by the acetylome. **Table S4.** Enrichment of DEPs and DAPs. **Table S5. **Content of lipids in the lipidome.

**Additional file 2:** **Fig. S1.** Differential analysis among three groups. **A** Volcano plot of DEGs between different groups. Blue denotes downregulated proteins, and red denotes upregulated proteins. **B** Number of up- and downregulated DEGs between different groups. **C** Heatmap of DEGs via hierarchical cluster analysis. Different columns correspond to different genes, and red and blue strips represent up- and downregulation, respectively. **D** and **E** Heatmap of DEPs (D) or DAPs (E) via hierarchical cluster analysis. Different columns correspond to different proteins, and orange and green strips represent up- and downregulated DEPs or hyper- and hypoacetylated DAPs, respectively. **F** Numbers of up- and downregulated DEPs between different groups. **G** Numbers of hyper- and hypoacetylated sites and proteins between different groups. **H** Volcano plot of DEPs between different groups. Green denotes downregulated proteins, and orange denotes upregulated proteins. **I** Volcano plot of DAPs between different groups. Green denotes downregulated proteins, and orange denotes upregulated proteins.

**Additional file 3:** **Fig. S2.** Comprehensive analysis of hepatic steatosis. **A** PPI network of DEPs and DAPs between HS0 and HS2. The green circles represent the downregulated DEPs, the yellow circles represent the upregulated DEPs, the purple circles represent the hypoacetylated proteins, and the red circles represent the hyperacetylated proteins. **B** Soft cluster analysis and enrichment analysis of the globe acetylated proteins. **C** Relative acetylated expression of K306 in MDH2. **D** Protein expression of SIRT3 in different groups. **E** CE/FC ratios in different groups. The values are the mean ± SEM. * represents *P* <0.05, ** represents *P* < 0.01 and *** represents *P* < 0.001.

## Data Availability

All data generated or analyzed during this study are included in this published article (and its supplementary information files).

## Abbreviations

CE: Cholesterol esters
CM: Chylomicrons
DAP: Differentially acetylated proteins
DEG: Differentially expressed genes
DEP: Differentially expressed proteins
DG: Diglycerol
DNL: De novo lipogenesis
FC: Free cholesterol
FFA: Free fatty acid
GO: Gene ontology
H&E: Hematoxylin and eosin
HDL: High-density lipoproteins
HS: Hepatic steatosis
KEGG: Kyoto encyclopedia of genes and genomes
MAFLD: Metabolic associated fatty liver disease
NAFLD: Nonalcoholic fatty liver disease
PC: Phosphatidylcholine
PCA: Principal component analysis
PE: Phosphatidylethanolamine
PPAR: Peroxisome proliferator-activated receptor
PPI: Protein‒protein interactio
PTM: Posttranslational modification mechanism
TBA: Total bile acid
TCA: Tricarboxylic acid cycle
TG: Triglyceride
VIP: Variable importance of the projection
VLDL: Very low-density lipoproteins
