# Supplementary figures and images for: Hepatic steatosis is associated with dysregulated cholesterol metabolism and altered protein acetylation dynamics in chickens
Source: J Anim Sci Biotechnol. 2023 Aug 12;14:108. doi: 10.1186/s40104-023-00910-8 (PMC10422840; doi:10.1186/s40104-023-00910-8)

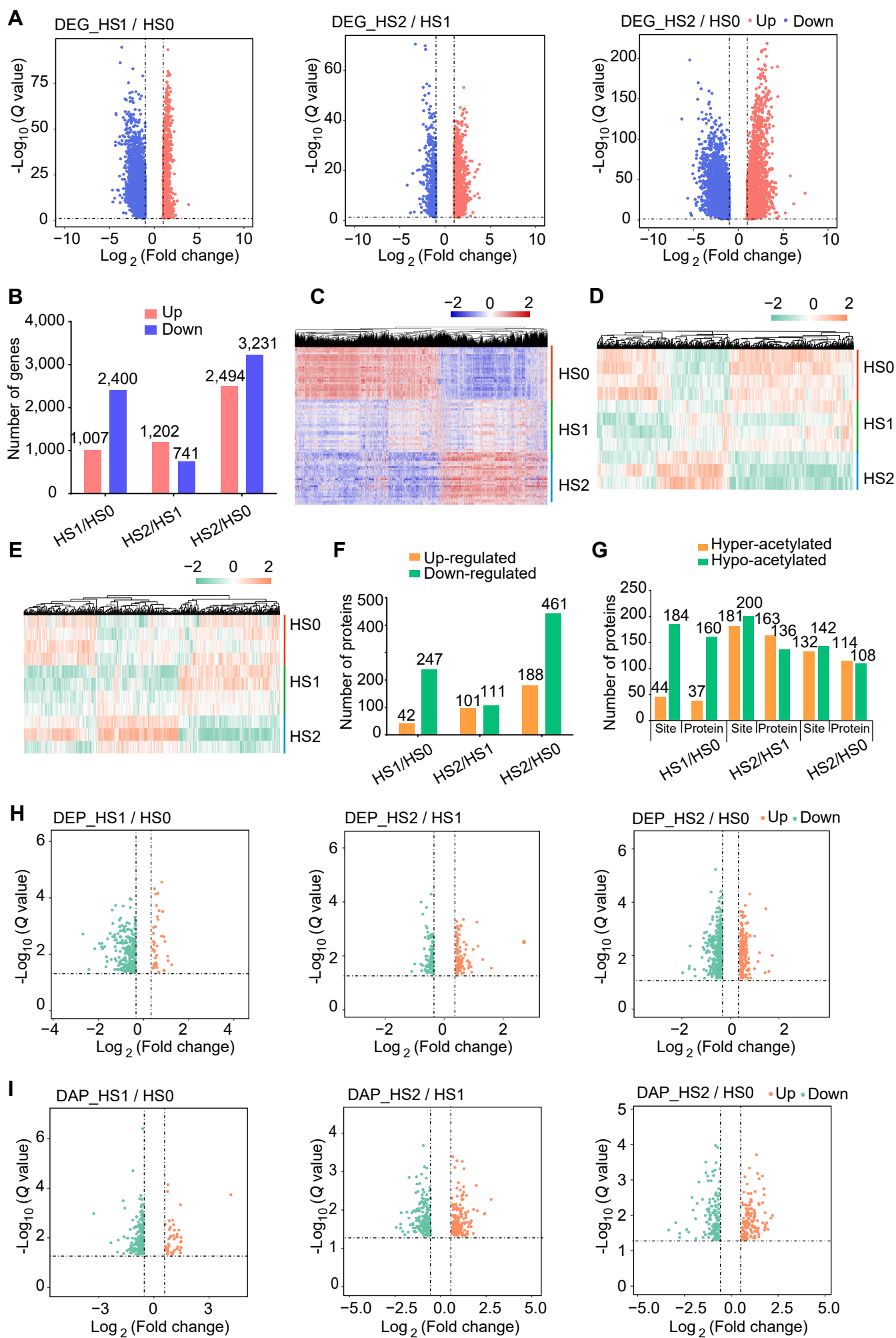

Supplement: Supplementary file 2 — Additional file 2: Fig. S1. Differential analysis among three groups. A Volcano plot of DEGs between different groups. Blue denotes downregulated proteins, and red denotes upregulated proteins. B Number of up- and downregulated DEGs between different groups. C Heatmap of DEGs via hierarchical cluster analysis. Different columns correspond to different genes, and red and blue strips represent up- and downregulation, respectively. D and E Heatmap of DEPs (D) or DAPs (E) via hierarchical cluster analysis. Different columns correspond to different proteins, and orange and green strips represent up- and downregulated DEPs or hyper- and hypoacetylated DAPs, respectively. F Numbers of up- and downregulated DEPs between different groups. G Numbers of hyper- and hypoacetylated sites and proteins between different groups. H Volcano plot of DEPs between different groups. Green denotes downregulated proteins, and orange denotes upregulated proteins. I Volcano plot of DAPs between different groups. Green denotes downregulated proteins, and orange denotes upregulated proteins. [file 40104_2023_910_MOESM2_ESM.pdf]

**A**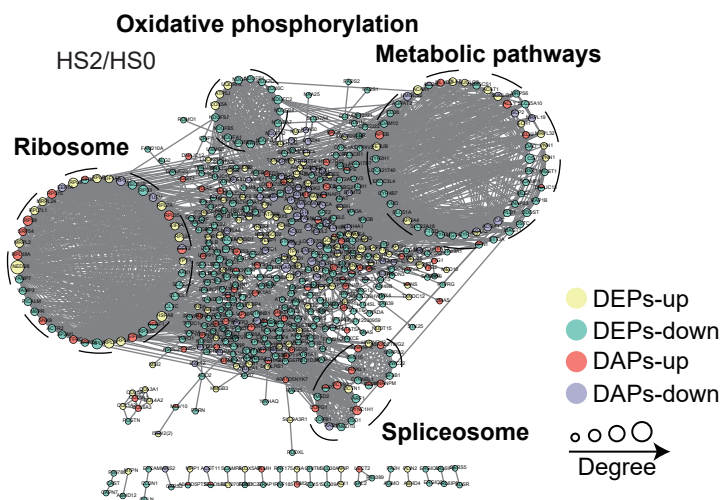**B**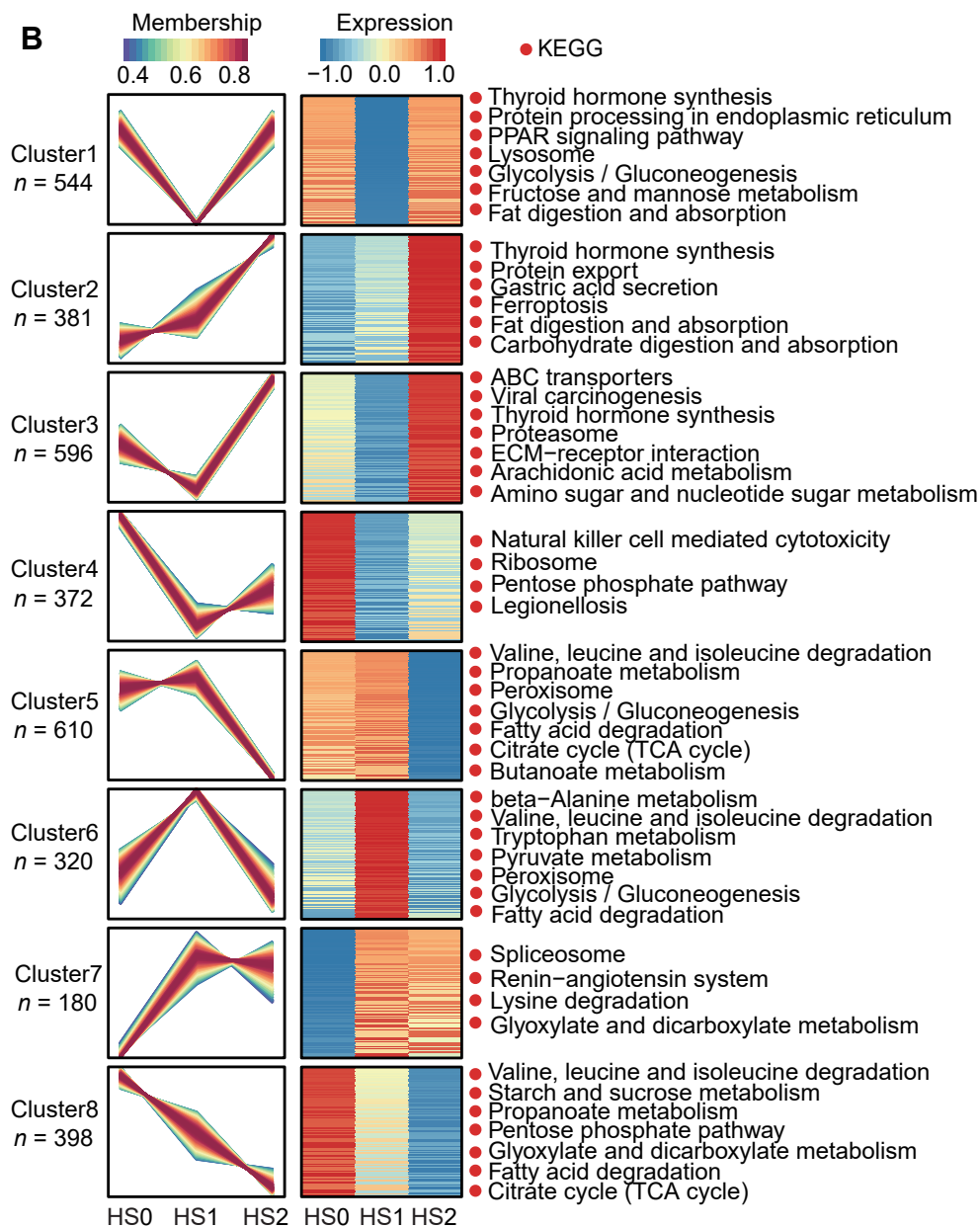**C**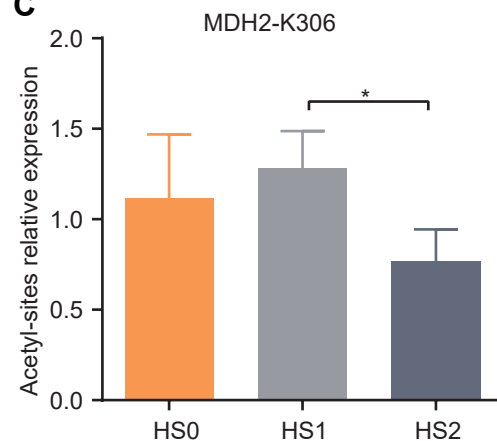**D**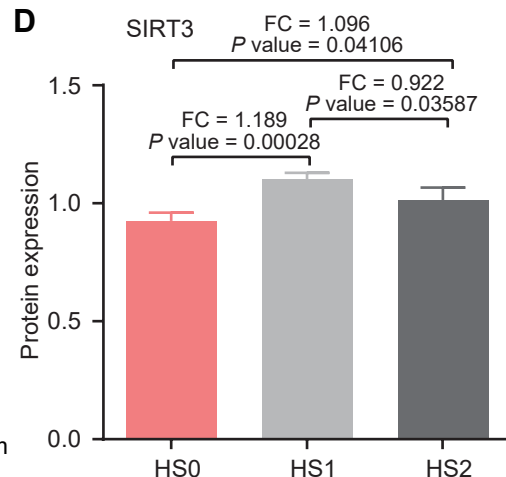**E**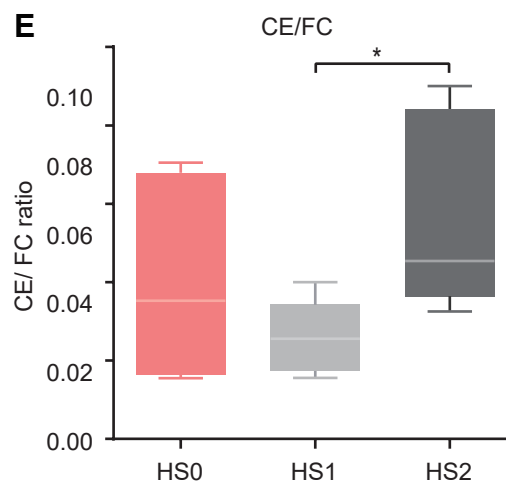

Supplement: Supplementary file 3 — Additional file 3: Fig. S2. Comprehensive analysis of hepatic steatosis. A PPI network of DEPs and DAPs between HS0 and HS2. The green circles represent the downregulated DEPs, the yellow circles represent the upregulated DEPs, the purple circles represent the hypoacetylated proteins, and the red circles represent the hyperacetylated proteins. B Soft cluster analysis and enrichment analysis of the globe acetylated proteins. C Relative acetylated expression of K306 in MDH2. D Protein expression of SIRT3 in different groups. E CE/FC ratios in different groups. The values are the mean ± SEM. * represents P <0.05, ** represents P < 0.01 and *** represents P < 0.001. [file 40104_2023_910_MOESM3_ESM.pdf]
